# Developing and Implementing ICF-Based Tools for Occupational Rehabilitation Supporting the Communication and Return to Work Process Between Sickness Absentees, Clinical Team and Jobcentre Contacts

**DOI:** 10.3389/fresc.2022.830067

**Published:** 2022-07-15

**Authors:** Thomas Johansen, Astrid Marie Kvaal, Ása Dóra Konráðsdóttir

**Affiliations:** ^1^Norwegian National Advisory Unit on Occupational Rehabilitation, Rauland, Norway; ^2^Department of Health and Social Services, Vinje Municipality, Vinje, Norway; ^3^Hæfi Rehabilitation Center, Reykjavík, Iceland

**Keywords:** ICF core set, occupational rehabilitation, work ability, sick leave, return to work (RTW), functioning

## Abstract

**Background:**

The ICF model is applied as a conceptual framework in occupational rehabilitation in Norway.

**Objective:**

To systematically apply the ICF model in rehabilitation this study had the following aims: (1) apply an ICF subset by merging an ICF core set and an ICF set to assess functioning in rehabilitation patients related to work; (2) develop a patient-reported ICF questionnaire and a clinician-friendly ICF report complementing the clinician-rated ICF subset and (3) evaluate whether ICF-based tools (subset, questionnaire, report) support the communication between a clinical team, patient and jobcentre contacts during return to work (RTW) follow up.

**Methods:**

Forty-one patients completing four weeks rehabilitation were recruited. The patients were referred from general practitioners and jobcentres. The ICF subset was a combination of the EUMASS core set for disability evaluation and suggested ICF categories by experts in vocational rehabilitation from Iceland. A clinical rehabilitation team interviewed the patients using the ICF subset and problems were quantified on a generic qualifier scale for body functions, activities and participation and environmental factors. The research team and clinical team developed an ICF questionnaire, by cross-culturally adapting the Work Rehabilitation Questionnaire to Norwegian. The same teams also developed an ICF report. The rehabilitation clinic forwarded the report and questionnaire to the patients' jobcentre contact, which was responsible for the RTW follow up. To evaluate the benefits of ICF-based tools, the clinical team, user representative and jobcentre contacts together participated in four workshops. They were asked the degree to which and in what way the tools supported the communication between them.

**Results:**

The ICF subset captured RTW challenges but was found to be time consuming. The jobcentres experienced the ICF report and questionnaire beneficial in the follow up as it strengthened their RTW decision-making basis and communication with the rehabilitation clinic and the patients about follow-up interventions.

**Conclusion:**

The development and implementation of ICF-based tools for clinical practice was a preliminary success in supporting the communication between three stakeholders during RTW follow up. Future applications of ICF-based tools ought to integrate personal factors to capture both facilitators and barriers related to functioning and work, thus, getting closer to a holistic assessment.

## Introduction

The International Classification of Functioning, Disability and Health (ICF) is a classification and coding system based on a theoretical biopsychosocial model. The ICF reflects a holistic view of health, meaning that functioning and disability capture the biological and psychosocial aspects of health. One of the aims of the ICF is to provide a common language of functioning which all health professionals and patients can use (1). The ICF seeks to address health and functioning as a relationship between health condition, body functions and structures, activities, participation, environmental factors and personal factors (2). The latter are not classified in the ICF coding system mainly due to ethical aspects related to such factors as well as challenges reaching common factors based on societal and cultural diversity (3).

The ICF (2) and the Sherbrooke model, an ecological case-management model of work disability prevention (4) are the two models applied as frameworks in occupational rehabilitation in Norway (5). The case-management model is an operational model that emphasizes the importance of all stakeholders around the worker (personal, workplace, healthcare and compensation systems) that influence the return to work (RTW) and disability process. A key component in rehabilitation, highlighted in the Sherbrooke model, is collaboration with local stakeholders such as the workplace, and jobcentres responsible for the RTW follow up of rehabilitation patients (5). Assessing functioning and work ability, based on the ICF, may be fruitful to apply in occupational rehabilitation, thus complementing existing assessment procedures during rehabilitation related to work ability, RTW self-efficacy, RTW expectations, anxiety, depression and pain (6). This is also the case for jobcentres having their own work ability assessment because they are responsible for the follow up of sick-listed individuals, the target group of the present study. Each sickness absentee has their own case management worker supporting them in the RTW process.

The WHO has in collaboration with several research groups worldwide developed ICF core sets for a variety of health conditions and diagnoses (7). The main goal has been to operationalize the comprehensive ICF classification system, consisting of more than 1,400 ICF categories. An ICF core set refers to an extract of categories from the ICF classification that are relevant to assess for a given health condition. The ICF does not provide guidelines on how to apply the classification in clinical practice. However, the development of core sets is one way to promote the implementation of ICF in clinical practice. Systematic research has been invested in developing, testing and validating ICF core sets (8). In Norway, testing and validation of ICF core sets for low back pain (9, 10) and rheumatoid arthritis (11) have been conducted. Moreover, an ICF core set for disability evaluation related to functional assessments in social security benefits has been developed by the European Union of Medicine in Assurance and Social Security (EUMASS) where 11 countries participated (12). The ICF core set used in the present study was a combination of the EUMASS core set (12) and suggested ICF categories by experts in vocational rehabilitation in Iceland (13). Although the EUMASS core set and the vocational rehabilitation set did not adhere to the established ICF core set development process (7) they were developed through a rigorous and standardized consensus procedure (12, 13). Therefore, we use the term ICF subset referring to the fact that an ICF core set was not used in the present study. We wanted to take a broad approach and included categories that would capture functioning related to work in both short and long term sick-listed individuals (14), hence we decided to combine the two sets that together covered the biomedical, social and psychological aspects of a person's lived experience of health. Long term sick leave was in the present study defined as sickness absence of more than six weeks (15, 16).

The ICF is unique in the sense that it is generic and that disability related to both work and non-work settings can be compared. That is why this study attempted to establish a collaboration between three key stakeholders responsible for RTW, namely, rehabilitation patients, clinical team in rehabilitation and jobcentre contacts, all emphasizing personal factors of will and goal, complying with the strong focus on goal setting in rehabilitation. It was guided by a process model in occupational rehabilitation, involving several stakeholders having emphasis on goal setting and the coordination of services (17) as well as recommendations to standardize the use of ICF for clinical practice (18). The aim of the present study was threefold to take into account the process perspective with regards to RTW: (1) apply an ICF subset, by merging one already established ICF core set and another ICF set, to assess the functioning in rehabilitation patients related to work; (2) develop a patient-reported ICF questionnaire and a clinician-friendly ICF report complementing the ICF subset assessment; and (3) evaluate whether ICF-based tools (subset, questionnaire, report) support the communication between a clinical team, patient and jobcentre contacts in RTW follow up.

## Methods

### Participants

#### Occupational Rehabilitation Patients

The patients were recruited from an occupational rehabilitation clinic in the specialist health care service, serving the South-East of Norway. Inclusion criteria were: aged between 18 and 67 and completing a 4-week inpatient rehabilitation programme. The patients were referred for rehabilitation by general practitioners or jobcentres (sickness absence insurance offices). Before referral to the rehabilitation programme, appropriate medical and work-related interventions had been attempted in the primary health care service, and thus, the patients required more comprehensive rehabilitation to be able to RTW. The patients attended individual and group-based interventions aiming to improve work ability, functioning related to work and goal setting for future work participation. Specific interventions included physical activity, making a written RTW plan, cognitive treatment components based on principles from cognitive therapy, acceptance and commitment therapy, psychoeducation, and motivational interviewing, and collaboration with the employer and the jobcentre (19).

#### User Representative

A former patient having previously completed occupational rehabilitation and who was working closely with a national rehabilitation user organization ensured user involvement. The user representative was systematically involved in developing the ICF questionnaire and ICF report.

#### Clinical Occupational Rehabilitation Team

The interdisciplinary clinical team comprising a physician, physiotherapist, work consultant, psychiatric nurse, psychologist and sports pedagogue performed the clinician-rated assessments (Table 1).

**Table 1:** Overview of stakeholder involvement.

**Stakeholder**	**ICF-based tool**	**What did the stakeholder do?**
Clinical team	ICF subset	Assigned problem (body functions), problem (performance) (activities and participation), barrier (environmental factors) qualifiers
Clinical team	ICF report	Summarized results from subset and questionnaire
Rehabilitation patient	ICF questionnaire	Completed the patient-reported questionnaire
Jobcentre contact	ICF report, ICF questionnaire	Used the results in the report and questionnaire to decide upon appropriate interventions during RTW follow up

#### Research Team

Two research institutions participated each having one researcher in the working group (TJ and ÁDK).

#### Jobcentres

Six jobcentres located in the South-East of Norway participated. The jobcentres apply their own work ability assessment, which is conducted as a semi-structured interview addressing barriers and facilitators for RTW, education, interests, personal goals, social circumstances and health. This assessment is also carried out to determine the degree to which the worker is entitled to health-related benefits, such as sick leave benefits or disability benefits (20).

### Materials and Procedure

#### Clinician-Rated ICF Subset

Prior to using the ICF subset in clinical practice, the clinical team received extended training in administering the subset by the research collaborator (ÁDK). This included a presentation on ICF, how human functioning is conceptualized through the ICF model and its classification system. The hierarchical arrangement of ICF components, chapters and level categories was also studied in detail. The training also included the usage of the generic ICF qualifiers, which quantify the extent of a problem experienced by a person in a specific ICF category (2). For body functions and activities and participation the qualifiers from 0 to 4 were used (0 = no problem, 1 = mild problem, 2 = moderate problem, 3 = severe problem, 4 = complete problem). The performance qualifier was used for activities and participation. For the environmental factors there are nine response categories that can either be facilitator or barrier (+4 = complete facilitator, +3 = substantial facilitator, +2 = moderate facilitator, +1 = mild facilitator, 0 = neither barrier nor facilitator, 1 = mild barrier, 2 = moderate barrier, 3 = severe barrier, 4 = complete barrier). The response options “8 – not specified” and “9 – not applicable” were used when appropriate.

The clinical team used the qualifiers and assessed the patients when commencing rehabilitation (pretest) and at the end of rehabilitation (posttest). The assessment was based on a semi-structured interview. First, in a consensus meeting prior to assessing patients, the six clinical team members were assigned ICF categories according to their domain of expertise. For example, all team members were assigned to assess attention functions (b140), while sensation of pain (b280) was assigned to the physician and physiotherapist, and school education (d820) was assigned to the work consultant. This assignment of qualifiers was adopted because the collaborating partner in Iceland had positive experiences in assigning domain specific qualifiers to ensure a competence-based assessment. Second, during the assessment of patients, each clinician individually assigned qualifiers on their own before discussing the individual ratings with the other team members. Consensus was reached where there were discrepancies.

The ICF subset used in the present study is displayed in Table 2, and comprised the EUMASS core set for functional assessments in disability evaluation (20 categories) (12) and the set for vocational rehabilitation developed in Iceland (13 categories) (13). The latter is different from the brief ICF core set for vocational rehabilitation (21). The EUMASS core set was developed through a formal decision-making process, where national EUMASS experts first suggested ICF categories and thereafter members of a working group voted on which ICF categories to be included in the final core set (12). The ICF categories from Iceland were also developed through a formal decision-making process where national experts in vocational rehabilitation first suggested ICF categories to be evaluated in vocational rehabilitation and thereafter a working group of physicians, physical therapists, occupational therapists, psychologists and social workers reached a final consensus on the included categories (13).

**Table 2:** ICF subset categories for occupational rehabilitation (*n* = 33).

**ICF code**	**Category title**	**Origin**
	**Body functions**	
b130	Energy and drive functions	Vocational rehabilitation Iceland
b134	Sleep functions	Vocational rehabilitation Iceland
b140	Attention functions	Vocational rehabilitation Iceland
b152	Emotional functions	Vocational rehabilitation Iceland
b164	Higher-level cognitive functions	EUMASS disability evaluation
b280	Sensation of pain	EUMASS disability evaluation
b455	Exercise tolerance functions	EUMASS disability evaluation
b710	Mobility of joint functions	EUMASS disability evaluation
b730	Muscle power functions	EUMASS disability evaluation
	**Activities and participation**	
d110	Watching	EUMASS disability evaluation
d115	Listening	EUMASS disability evaluation
d155	Acquiring skills	EUMASS disability evaluation
d177	Making decisions	EUMASS disability evaluation
d220	Undertaking multiple tasks	EUMASS disability evaluation
d240	Handling stress and other psychological demands	EUMASS disability evaluation
d399	Communication, unspecified	EUMASS disability evaluation
d410	Changing basic body position	EUMASS disability evaluation
d415	Maintaining a body position	EUMASS disability evaluation
d430	Lifting and carrying objects	EUMASS disability evaluation
d440	Fine hand use	EUMASS disability evaluation
d445	Hand and arm use	EUMASS disability evaluation
d450	Walking	EUMASS disability evaluation
d470	Using transportation	EUMASS disability evaluation
d570	Looking after one's health	Vocational rehabilitation Iceland
d720	Complex interpersonal interactions	EUMASS disability evaluation
d760	Family relationships	Vocational rehabilitation Iceland
d820	School education	Vocational rehabilitation Iceland
d850	Remunerative employment	Vocational rehabilitation Iceland
d870	Economic self-sufficiency	Vocational rehabilitation Iceland
d920	Recreation and leisure	Vocational rehabilitation Iceland
	**Environmental factors**	
e310	Immediate family	Vocational rehabilitation Iceland
e460	Societal attitudes	Vocational rehabilitation Iceland
e580	Health services, systems and policies	Vocational rehabilitation Iceland

#### Patient-Reported ICF Questionnaire

The Work Rehabilitation Questionnaire (WORQ), an ICF-based instrument for vocational rehabilitation, has been validated to assess functioning in vocational rehabilitation (22). The patient-reported WORQ is a derivative of the ICF core set for vocational rehabilitation (21). WORQ comprises two parts: part one sociodemographics and part two ICF-based items. For the present study it was appropriate to cross-culturally adapt part two of the WORQ self-reported English version into Norwegian as 33 of WORQ's 42 items were identical with the categories in the ICF subset (Table 2). Existing instruments already applied in rehabilitation covered part one (sociodemographics). The general recommendation for cross-cultural translation was followed in the adaptation process (23) with some modifications to integrate learned lessons from administering the ICF subset. The forward translation was conducted by three translators of which one was bilingual in English. All translators were aware of the purpose of the questionnaire and their backgrounds were psychology, physiotherapy and nursing. The three translated questionnaires were compared and a questionnaire synthesized from the translation of the three translators, resolving discrepancies between the versions, was developed. The synthesis version of the ICF questionnaire was pre-tested on nine patients to investigate its user friendliness, wording and verbal feedback given by each patient. The time taken to complete the pre-test version of the questionnaire and give feedback to the examiner was 15–30 min.

#### Clinician-Friendly ICF Report

The research team, clinical team and jobcentres developed an ICF report to systematically follow up patients who did not return to full time employment following rehabilitation. In the report, the clinical team summarized the clinical and patient-reported findings of functioning and work ability from the clinician-rated ICF subset assessment, patient-reported ICF questionnaire assessment and other standardized assessments carried out during rehabilitation. This summary of findings was used to provide the jobcentre contacts with individual patient specific information on functioning and work ability, and on that basis, suggest specific RTW interventions to be discussed between the contact and the patient during the RTW follow up period. The collaborating stakeholders structured the report according to the ICF components including personal factors and goal setting. The report is included as Supplementary Material. Thus, the content of the report was divided into four sections: (1) summary of functioning and work ability assessments carried out prior to rehabilitation by the employer, general practitioner and jobcentres, (2) patient-related RTW goals during rehabilitation, (3) summary of functioning and work ability according to ICF subset and ICF questionnaire for activities and participation, personal factors, environmental factors including RTW facilitators and barriers based on physical and psychological demands in current work, and (4) suggested work-related interventions in the primary health care service, specialist health care service or by employer and jobcentres following rehabilitation. On the final day of rehabilitation, the patient read through and approved the content of the ICF report prior to sending it to the patient's jobcentre contact.

### ICF Workshops

An overview of ICF workshops is given in Table 3 where the topic of the workshops and the participating stakeholders are presented in chronological order during the 2-year study period. The workshops were designed to facilitated knowledge transfer and exchange between the stakeholders. There were three project phases. Phase 1 (pre-project phase) included supervisory guided ICF training to ensure that all clinicians in the team were at the same level with regards to ICF knowledge and competencies. A session was also devoted to assigning ICF subset qualifiers according to each clinicians' area of expertise to ensure that each qualifier was scored and evaluated based on optimal clinical knowledge. This was followed by a workshop where clinicians discussed the scoring and consensus procedure in the ICF subset assessments. Phase 2 (project phase) included workshops devoted to identify the common language between the ICF subset assessment and the work ability assessment used by the jobcentres, cross-cultural adaptation of the 33 items from the WORQ to Norwegian to produce the ICF questionnaire, development of the ICF report and usage of common language to better communicate ICF results to the jobcentres. Progression was ensured during weekly supervision. Phase 3 (learning evaluation during and after project period) focused on what the stakeholders had learned and achieved and what would be the preferred learning outcome at the end of the project period. The learning evaluation conducted after the project period focused on implications for clinical practice and implementation of results in clinical practice. A workshop was also devoted to reaching consensus on the common ICF language in the report contributing to improved communication between the clinical team, patient and jobcentre. Here the clinical team, patient and jobcentres were specifically asked the degree to which and in what way the tools supported the communication between them. The written content of each workshop was summarized and distributed among the participating stakeholders (research team, clinical team, user representative, jobcentres).

**Table 3:** Chronological order of ICF workshops and topics including the participating stakeholders [research team (RT), clinical team (CT), Jobcentre (JC), user representative (UR)].

	**Chronological order of ICF workshops**	**Participating stakeholders**				**Topic of workshop**
Phase 1	2-Day preparation workshop prior to project start	RT	CT			- Supervisory guided ICF training of classification, model, qualifiers, components, codes and category definitions of the ICF subset - Team training in administering the ICF subset
		ICF category assignment	RT	CT			- Each clinician assigned ICF categories according to area of expertise
		ICF related project issues	RT	CT			- Supervision of the ICF subset assessment - Two weekly meetings during project period between research team/clinical team/jobcentre
		1-Day ICF core set workshop	RT	CT	JC		- Scoring and consensus procedure in ICF subset assessment
Phase 2	1-Day preparation workshop	RT	CT	JC	UR	- Supervisory guided ICF training in collaboration with jobcentre - Identification of common language between ICF subset and work ability assessment instrument from jobcentre supporting the communication between stakeholders
		ICF questionnaire	RT	CT	JC	UR	- Cross-cultural adaptation (using 33 of 42 Work Rehabilitation Questionnaire items)
		1-Day ICF core set workshop	RT	CT	JC		- Identification of common language supporting the communication of ICF results to jobcentres
		ICF report	RT	CT	JC	UR	-Development of report -Report structured according to ICF model and work ability assessment from jobcentre
Phase 3	1-Day learning evaluation workshop	RT				- What have I/my organization learned/achieved from the collaboration? - My organization is/I am so pleased at the end of the project period because?
		1-Day learning evaluation workshop	RT		JC		Key reflection statements: - ICF questionnaire support the follow up because… - ICF report contains sufficient information during follow up because… - Jobcentres should be involved prior to rehabilitation because… -Jobcentres can merge the ICF report with the work ability assessment because… - ICF report could be improved in the following way… - My main message to the research team is…
		1-Day learning evaluation workshop	RT	CT			- Implications for clinical practice
		2-Day final evaluation workshop	RT	CT	JC	UR	- Reach consensus on common language in report - Implications for clinical practice and implementation of project results in clinical practice

### Study Design

In the first phase of the study, the clinical team administered the ICF subset assessment at posttest. In the second phase, the subset was administered at the timepoints pretest and posttest to capture the degree of changes during rehabilitation in functioning and work ability. The application of the patient-reported ICF questionnaire followed the same phases. First administration at posttest followed by pretest and posttest assessments. The ICF report was completed by the clinical team at posttest. Posttest assessments were carried out first because the team needed to get experience in reporting on the patients' functioning and work ability to the jobcentres. To provide the jobcentre contacts with information on the degree of changes in functioning and work ability the assessments were also conducted at pretest and posttest for the ICF subset and ICF questionnaire. The period between the pretest and posttest was 4 weeks.

## Results

### Sample Characteristics

The total sample included 41 patients of which 60% were on partial sick leave and 40% were on full time sick leave (28 female, 13 male, mean age = 47, standard deviation 6.5). The mean length of sick leave prior to rehabilitation was 23 weeks. Full time sick leave refers to 100% sickness absence whereas partial sick leave is any graded sickness absence below 100%.

### Clinician-Rated ICF Subset

The ICF subset assessment of each patient took ~10–20 min and the duration of the consensus meeting in the clinical team for each patient lasted 30–60 min. The clinical team assessed the patients to have problems in all 33 ICF categories with a frequency from 5 to 95%. The five most frequently scored problem categories in body functions and activities and participation and the two most frequently scored barrier categories in environmental factors are displayed in Table 4. A problem and a barrier were defined if the clinical team assigned a qualifier between 1 and 4. The clinical team found the subset assessment to capture the patients' functioning, work ability and return to work challenges but was experienced as time consuming.

**Table 4:** The five most frequently scored problem categories by the clinical team (qualifiers 1, 2, 3 and 4) in body functions and activities and participation and the two most frequently scored barrier categories in environmental factors related to functioning and work ability at posttest (*n* = 23).

**ICF categories**	**% of patients**
	**with problems**
**Body functions**	
b130 Energy and drive functions	96
b134 Sleep functions	91
b152 Emotional functions	87
b280 Sensation of pain	87
b455 Exercise tolerance functions	74
**Activities and participation**	
d240 Handling stress and other psychological demands	100
d920 Recreation and leisure	100
d570 Looking after one's health	96
d220 Undertaking multiple tasks	74
d850 Remunerative employment	63
**Environmental factors**	
e310 Immediate family	70
e460 Societal attitudes	35

### Patient-Reported ICF Questionnaire

The 33 ICF categories shown in Table 2 were adapted to Norwegian from the WORQ. The research team and clinical team agreed that the ICF subset mainly focused on barriers and addressed this issue during the development of the ICF questionnaire, shifting the focus from barriers to facilitators. This followed from the feedback from the patients and the examination of the translated version of the ICF questionnaire by the user representative, research team, clinical team and each of the six contact persons at the jobcentres. It was therefore decided to frame the ICF items in the questionnaire positively.

Example question from the adapted ICF questionnaire: Item b730, ≪During the past four weeks, to what extent have you… …had enough muscle strength to carry out your daily activities≫ Response options: ≪0 = to a very small extent≫ to ≪10 = to a very large extent≫. The recall period was changed from one to four weeks corresponding to the length of rehabilitation. These modifications to items and recall period were carried out to increase the applicability of the questionnaire in the current rehabilitation context and do not adhere to the design and content of the standardized WORQ.

### Clinician-Friendly ICF Report

The time taken to complete the ICF report by the clinical team for each patient took ~20–30 min. In total, the clinical team and the research team completed a report on 11 rehabilitation patients who all read their individual ICF report and consented to sending the report to their local jobcentre contact responsible for the RTW follow up. The jobcentres found the ICF questionnaire and the ICF report beneficial in the follow up of patients after rehabilitation as it strengthened their RTW decision-making basis and communication with the clinical team and the patient about further work-related interventions.

### ICF Workshops

The workshops facilitated knowledge transfer and exchange during the study period and after study completion. The clinical team and the jobcentre contacts emphasized that the main learning outcome at the end of the study period was the adoption of new ways of working and collaborating, based on the ICF, between the clinical team, patients and jobcentre contacts (see also Table 3 for an overview of topics covered during the workshops).

## Discussion

### Summary of Findings

The results of this study showed that the collaborating stakeholders, a clinical team, patients and jobcentre contacts, found the ICF subset not suitable to be administered in clinical practice on its own and therefore supplementary tools were needed to carry out a holistic assessment during occupational rehabilitation. This led to the development of a patient-reported ICF questionnaire and clinician-friendly ICF report supporting the clinician-rated ICF subset assessment. These tools were found to be beneficial in the communication between the clinical team, patients and jobcentre contacts during the RTW follow up period after rehabilitation. It was a step in the direction of reaching a common language based on the ICF, supporting the communication between the clinical team and the jobcentres and between the patients and the jobcentres. The jobcentres argued that the report and questionnaire gave them a stronger foundation to make decisions about further work-related interventions for RTW seeing facilitators and barriers together to capture a holistic perspective on the opportunities for RTW.

### ICF Training

Supervisory guided training to increase the knowledge about the ICF classification, its coding system and the rationale for developing and administering ICF core sets was conducted prior to using the ICF-based tools. We suggest this to be mandatory for all clinicians intending to use ICF-based tools. Ideally the supervisor should be a clinician with extensive training and experience in using and applying the ICF in clinical practice, such as in the present study. It seems fruitful to dedicate one or two clinicians who receive extended training in using the ICF and are responsible for collaborating with stakeholders during rehabilitation, and in the RTW follow up process (24).

### ICF Subset Assessment

The clinical team experienced challenges in using the ICF subset in clinical practice. The assessment was time consuming, taking at least 40 min for each patient. The time consuming administration procedure was a barrier for implementing the ICF subset in a Norwegian occupational rehabilitation setting. These findings corroborate other studies applying ICF core sets in clinical practice (9, 10). Furthermore, using ICF core sets in clinical practice has been identified as challenging (25). Which of the 1,400 ICF categories are suitable for assessment, given a specific health condition? And how do we ensure that clinicians have the expertise and competence required to administer core sets? Personal factors, which is a major component in rehabilitation, tend not to be linked to goal setting in the ICF (26). Having said this, the WHO has emphasized that the ICF is a terminology and a classification system, and not a measurement instrument. The terminology can be used to develop an instrument and existing instruments can be mapped to ICF terminology, such as the WORQ (22). The ICF categories in the subset are assumed to be relevant for occupational rehabilitation because we combined the core set for disability evaluation (12) and the set for vocational rehabilitation (13). Therefore, the ICF subset assessment did capture relevant facilitators and barriers for RTW, but the clinicians argued that the patient perspective was lacking. The clinicians also stated that it was unsuitable as a communication tool between the jobcentre contacts and the patients. This laid the foundation for integrating the three ICF-based tools and will be further discussed and elaborated upon.

### Integration of ICF-Based Tools in Rehabilitation

The clinical team found the combination of using the ICF subset, the ICF questionnaire and summarizing the findings in the ICF report beneficial for the patient because the jobcentres experienced the information in the ICF report highly relevant. The workshops contributed to maintain effective communication between the stakeholders and to develop a common understanding of RTW facilitators and barriers based on the ICF. And further, to improve competencies about the application of ICF-based tools in clinical practice and at the jobcentres. It can be argued, based on the discussions in the workshops, that the usage of ICF-based tools was partly successful in operationalising the ICF model and creating a common language that supported the communication between the clinical team, patient and jobcentres. It made the communication in the ICF report more efficient because it was founded on the language and content of the jobcentre's work ability assessment. The work ability assessment from the jobcentres can be viewed as a static assessment because it provides a cross sectional glimpse into work ability, whereas the ICF report is a dynamic report based on a 4-week rehabilitation programme taking into account actions relevant in the RTW follow up process. Therefore, the report was found to strengthen the decision making of the jobcentres, where specific follow up interventions were suggested for each patient. The content of the report was also synchronized with the aims of rehabilitation, namely, the focus on person-centered functioning such as coping, work-related self-efficacy, RTW expectations, experiences and resources (27–29). The development and application of the ICF-based tools seem to have resulted in an extended understanding of functioning and work ability, thus having the focus on salutogenic factors in the personal and activities and participation domains. Specifically, the positive framing of the items in the ICF questionnaire, may have contributed to focus not only on barriers but also on facilitators by all three stakeholders. Complementing the ICF qualifier approach by framing the ICF stem question positively could encourage reflections around empowerment and RTW self-efficacy (27). We believe that the framing modification was important due to the impact ICF have, and will have, on clinical practice (18, 30). The usage of ICF-based tools in the present study contributed to making ICF more applicable for clinical practice as well as during RTW follow up (18) where the focus was on opportunities for RTW, improving work ability, RTW expectations and RTW self-efficacy (6).

Sickness absence and work disability is a focus within the domain of activities and participation according to the ICF model (31). Occupational rehabilitation requires a relational approach between contextual factors both at home and work, with an emphasis on work participation. Successful RTW is more likely to be achieved if stakeholders in all system levels are involved (32). Focusing only on the individual is too narrow because a worker with a disability is dependent on the workplace, legislation and context. Still, the key challenge is knowing how and when to intervene in activities and participation and environmental and personal factors (17, 33). The present study was guided by the pragmatic application of the conceptual ICF model in occupational rehabilitation where the following questions are posed (17): (1) How should we describe functioning based on facilitators and barriers? (2) Which goals should be targeted in the rehabilitation process? (3) Which interventions support the goals? (4) Who is responsible for coordinating the services? These open-ended questions underpins the importance of approaching rehabilitation in terms of a process involving key stakeholders. ICF core sets guide clinicians to look at functional items that are often relevant for a particular group of patients and subsequently apply a process model. Applying a pragmatic approach seems to be an fruitful way forward contributing to holisitic assessments in rehabilitation (24). The usage of the conceptual ICF model, as the starting point of the current study, is consistent with the view that rehabilitation is about establishing an opportunity for participation according to individuals' desires and motivations (25, 26) and to enhance the subjective experience of human functioning despite a challenging health condition or disability (34).

### Strengths and Limitations

The strengths of this study were the usage of established ICF-based tools such as the EUMASS core set and WORQ as well as applying the ICF, which is an accepted international reference standard for operationalizing functioning. The description of the content of the ICF workshops was carried out to document the progress and reach consensus on the way forward to increase the standardization of the assessments. The main limitation was the lack of using ICF as a person-centered tool which was not fully captured in the semi-structured interview between the clinician and the patient. We did not use the ICF assessment sheet nor the rehabilitation problem-solving form which could have enhanced the assessments (35). To develop a thorough competency of ICF requires continuous and systematic work by clinicians and researchers, which was not the case for all clinical team members involved. The research team provided training if a new team member was not familiar with the ICF. However, this training was not considered comprehensive and may have negatively affected the quality of assessments. The user representative was only involved in developing the ICF questionnaire and ICF report, and systematic user involvement was not applied throughout. The results of the patient-reported ICF questionnaire and the clinician-rated ICF subset assessment must be considered exploratory, because of the small number of participants recruited. Further, it was decided to frame the questions in the ICF questionnaire positively, which contrasts with the framing in the WORQ. This change in psychometric properties should have warranted calculations of the instrument's internal consistency and inter-rater reliability using the intraclass correlation coefficient. Similarly, we did not carry out reliability or validity analyses on the ICF questionnaire because of the low number of participants recruited. More research is therefore needed to confirm the usefulness of the report and the questionnaire and the present results should be taken as exploratory.

## Conclusion

There is a need to develop and implement new and current assessments tools of functioning and work ability in occupational rehabilitation and the ICF-based tools developed in the current study is a step in that direction. The integration of the ICF subset, questionnaire and report was a preliminary success in creating a common language supporting the communication between a clinical team and six jobcentres and between the patient and the jobcentre contacts in the RTW follow up period. The result of the ICF subset assessment was deemed insufficient to communicate to the jobcentres during follow up of rehabilitation patients. To better fit with the work ability assessment language used by the jobcentres, ICF-based tools were developed. The jobcentres stated that the results in the report and the questionnaire laid the foundation for improved communication with the clinical team, enhanced the decision-making process where the jobcentre contact in dialogue with the patient could make informed decisions on appropriate interventions in the follow up period to increase the chances of RTW. Using ICF tools and include the person-centered focus in future clinical practice studies, should lay the foundations for a deeper understanding of the clinical and work-related implications of the ICF, underpinning holistic principles as well as making the ICF more applicable for clinical practice.

## Data Availability Statement

The data and documentation supporting the conclusions of this article will be made available from the corresponding author upon reasonable request.

## Ethics Statement

The participants were informed about the nature of the study and all gave written informed consent, which formed part of the standard procedure at the clinic prior to commencing rehabilitation. Therefore, it was not required to seek ethical approval for this study. Nevertheless, all procedures followed were in accordance with the ethical standards of the Helsinki Declaration.

## Author Contributions

TJ and ÁDK planned and designed the study. TJ and AMK were responsible for data collection. TJ carried out the analyses and wrote the first draft of the manuscript. AMK and ÁDK commented on and reviewed later versions of the manuscript. All authors read and approved the final manuscript.

## Funding

This study was funded by the Norwegian Labour and Welfare Administration.

## Conflict of Interest

The authors declare that the research was conducted in the absence of any commercial or financial relationships that could be construed as a potential conflict of interest.

## Publisher's Note

All claims expressed in this article are solely those of the authors and do not necessarily represent those of their affiliated organizations, or those of the publisher, the editors and the reviewers. Any product that may be evaluated in this article, or claim that may be made by its manufacturer, is not guaranteed or endorsed by the publisher.
