# Supplementary material for: Developing and Implementing ICF-Based Tools for Occupational Rehabilitation Supporting the Communication and Return to Work Process Between Sickness Absentees, Clinical Team and Jobcentre Contacts
Source: Front Rehabil Sci. 2022 Jul 15;3:830067. doi: 10.3389/fresc.2022.830067 (PMC9397679; doi:10.3389/fresc.2022.830067)
Supplement: Supplementary file 1 [file Table_1.pdf]

ARBEIDOGHELSE.NO
Norwegian National
Advisory Unit on
Occupational Rehabilitation

Rehabiliteringssenteret AiR
KLINIKK

ICF report to Jobcentre

**Name:**	**Rehabilitation period:**

This report is also based on results from the patient-reported ICF questionnaire

1. Before Rehabilitation:

- Information from employer on demands and expectations at work, e.g., job duties, job accommodations
  -
- Information from general practitioner on functioning and work ability
  -
- Information from jobcentre on functioning and work ability
  -

2. Goal setting during rehabilitation based on the ICF model (body functions, activities and participation, environmental and personal factors):

- Interdisciplinary assessment in week 1 of rehabilitation (patient and clinical team)
  - Goal 1: This goal should be work-related
  - Goal 2:
  - Goal 3:

3. Summary of assessments from the perspective of the individual, workplace and contextual factors:

- Functioning and work ability based on:
  - Activities and participation, personal factors, environmental factors, body functions

- Factilitators for RTW according to demands and expectations in current work
  - 1:
  - 2:
  - 3:

- Barriers for RTW according to demands and expectations in current work
  - 1:
  - 2:
  - 3:

- Goal setting after rehabilitation (elaborate if goals after rehabilitation is different to goals during rehabilitation
    - Goal 1:
    - Goal 2:
    - Goal 3:

4. Work-related interventions after rehabilitation – who does what:

- Worker:
- Jobcentre:
- General practitioner:
- Employer:
- Occupational health service:
- Work consultant at rehabilitation clinic:
- Other stakeholders:
